# Associations Between Sympathetic Nervous System Synchrony, Movement Synchrony, and Speech in Couple Therapy

**DOI:** 10.3389/fpsyg.2022.818356

**Published:** 2022-03-10

**Authors:** Anu Tourunen, Petra Nyman-Salonen, Joona Muotka, Markku Penttonen, Jaakko Seikkula, Virpi-Liisa Kykyri

**Affiliations:** ^1^Department of Psychology, Faculty of Education and Psychology, University of Jyväskylä, Jyväskylä, Finland; ^2^Department of Social Sciences and Philosophy, Faculty of Humanities and Social Sciences, University of Jyväskylä, Jyväskylä, Finland; ^3^Faculty of Health and Sport Sciences, University of Agder, Kristiansand, Norway; ^4^Faculty of Social Sciences, Tampere University, Tampere, Finland

**Keywords:** synchrony, couple therapy, electrodermal activity (EDA), MEA, speech, sympathetic nervous system, skin conductance, movement synchrony

## Abstract

**Background:**

Research on interpersonal synchrony has mostly focused on a single modality, and hence little is known about the connections between different types of social attunement. In this study, the relationship between sympathetic nervous system synchrony, movement synchrony, and the amount of speech were studied in couple therapy.

**Methods:**

Data comprised 12 couple therapy cases (24 clients and 10 therapists working in pairs as co-therapists). Synchrony in electrodermal activity, head and body movement, and the amount of speech and simultaneous speech during the sessions were analyzed in 12 sessions at the start of couple therapy (all 72 dyads) and eight sessions at the end of therapy (48 dyads). Synchrony was calculated from cross-correlations using time lags and compared to segment-shuffled pseudo synchrony. The associations between the synchrony modalities and speech were analyzed using complex modeling (Mplus).

**Findings:**

Couple therapy participants’ synchrony mostly occurred in-phase (positive synchrony). Anti-phase (negative) synchrony was more common in movement than in sympathetic nervous system activity. Synchrony in sympathetic nervous system activity only correlated with movement synchrony between the client-therapist dyads (*r* = 0.66 body synchrony, *r* = 0.59 head synchrony). Movement synchrony and the amount of speech correlated negatively between spouses (*r* = −0.62 body synchrony, *r* = −0.47 head synchrony) and co-therapists (*r* = −0.39 body synchrony, *r* = −0.28 head synchrony), meaning that the more time the dyad members talked during the session, the less bodily synchrony they exhibited.

**Conclusion:**

The different roles and relationships in couple therapy were associated with the extent to which synchrony modalities were linked with each other. In the relationship between clients and therapists, synchrony in arousal levels and movement “walked hand in hand”, whereas in the other relationships (spouse or colleague) they were not linked. Generally, more talk time by the therapy participants was associated with anti-phase movement synchrony. If, as suggested, emotions prepare us for motor action, an important finding of this study is that sympathetic nervous system activity can also synchronize with that of others independently of motor action.

## Introduction

Interpersonal synchrony in psychotherapy manifests in several modalities and seems to be important in shaping the client-therapist relationship and therapy outcome (Mende and Schmidt, 2021). However, a fundamental question remains: what is the association between the different synchrony modalities (Wiltshire et al., 2020)? We attempted to answer this question by studying the association between sympathetic nervous system synchrony, movement synchrony, and speech in couple therapy. Synchrony can be defined as individuals’ temporal coordination in social interactions. However, different but similar terms, such as mimicry, resonance, concordance, linkage, coupling, and attunement are used in different research fields (Delaherche et al., 2012; Palumbo et al., 2017). We have an automatic tendency to mimic others’ facial expressions, prosody, postures, movements, and behaviors, especially if we like someone and want to affiliate with them (van Baaren et al., 2004; Hatfield et al., 2009). Non-verbal synchrony can manifest in various modalities and levels, for example in body movement, autonomic nervous system (ANS) activity, brain activity (“hyperscanning”; Montague et al., 2002), or hormonal activity (“endocrine fit”; Feldman, 2017).

Synchrony is often beneficial in social situations, as it fosters prosocial behavior (Cirelli et al., 2014; Rennung and Göritz, 2016) and increases rapport in relationships (Hove and Risen, 2009; Miles et al., 2009; Lakens and Stel, 2011), although high synchrony is not automatically beneficial in all situations (Butler, 2015; Tourunen et al., 2020). In studies comparing experimental and control conditions, synchrony has been found to have a medium-sized positive effect on prosocial behavior, a small- to medium-sized positive effect on social bonding and social cognition, and a small-sized positive effect on positive affect (Mogan et al., 2017). In clinical psychology and psychotherapy, a better understanding of the functions of synchrony could lower drop-out rates, improve therapeutic interventions and reduce healthcare costs (Mende and Schmidt, 2021).

A good therapeutic alliance is considered one of the best predictors of outcome in individual psychotherapy (Orlinsky et al., 2004). It has been suggested that synchrony is related to this alliance by enabling access to another’s internal states via inter-brain coupling, which over time may improve emotional regulation and relate the therapy outcome (Koole and Tschacher, 2016). Most of the variance in therapy outcomes remains unexplained, and many researchers have suggested that the focus should be on how the mutual regulation between clients and therapists influences the change process (Safran and Muran, 2006). Traditional conceptualizations of the alliance may have overemphasized conscious rational collaboration while underestimating the importance of non-conscious factors, such as non-verbal synchrony. Relatedly, embodied cognition arose as a movement in cognitive science to accord the body a central role in shaping the mind (Wilson, 2002).

So far, studies have mainly focused on a single modality of synchrony without attending to the possible connections between different aspects of attunement. For example, movement can often be voluntarily controlled, whereas ANS activity is involuntary and reactions to internal and external events are highly automatic. The question then arises, does movement synchrony in therapy “tell the same story” as ANS synchrony? To our knowledge, this has not been studied in the context of therapeutic interactions, while the few experimental studies that exist have yielded conflicting results, suggesting that ANS synchrony and movement synchrony may be independent processes (Codrons et al., 2014; Noy et al., 2015) or that they may be connected in a bi-directional way (Zhang et al., 2016). Mayo and Gordon (2020) propose a flexible, adjusting model of two interpersonal tendencies: one toward synchronization and the other toward segregation, which act simultaneously and depend on the social context.

It is important to note that studies on synchrony have been conducted in very different research settings. In some settings, individuals have not been fully interacting; instead, e.g., one might only be watching the other (Konvalinka et al., 2011), individuals are watching a similar event (Nummenmaa et al., 2012; Ardizzi et al., 2020; Tschacher et al., 2021) or individuals may simply be in the same room without visual contact (Helm et al., 2012). This means that the results on synchrony, including those related to different modalities, tend to show wide diversity across different research settings and conditions (Palumbo et al., 2017). Group size is also likely to affect synchrony: for example, synchrony, especially in larger groups, can increase prosocial behavior and positive affect (Mogan et al., 2017). In the context of therapeutic encounters, it seems more reasonable to focus on settings that require participants to fully interact with each other.

### Sympathetic Nervous System Synchrony

Autonomic nervous system can be divided into the sympathetic nervous system (SNS), which prepares us for action by increasing arousal level (fight-or-flight response), and the parasympathetic nervous system (PNS), which is active during relaxation and recovery. One of the best ways to measure SNS activity is to record electrodermal activity (EDA) by means of skin conductance, as the eccrine sweat glands receive only excitatory sympathetic nerve impulses (Boucsein, 2012). An often-used measure of PNS activation is high frequency power in heart rate variability (Stein et al., 1994). Synchrony seems to exist between clients and therapists in both SNS and PNS measures; these in turn are associated with ratings of the therapy process (Tschacher and Meier, 2020). However, in this study we focused only on SNS synchrony via EDA, as it may not be as closely connected to movement as cardiac measures. The heart rate is more closely related to muscular action than EDA (Fowles, 1980), and speech affects the respiration rate, which in turn affects the frequency domain measurements of heart rate variability (HRV) via shifts in respiratory sinus arrhythmia (Schipke et al., 1999).

In individual psychotherapy, EDA synchrony has been associated with clients’ perceptions of feeling more empathy from their therapists (Marci et al., 2007). Emotional distance in these types of interactions has been associated with lower EDA synchrony and reduced ratings of perceived empathy (Marci and Orr, 2006). Messina et al. (2013) also found therapists to display more empathy and have more EDA synchrony with (pseudo)clients than psychologists or non-therapists.

In couple therapy, spouses score lower on the bond spectrum of alliance than clients in individual therapy (Bartle-Haring et al., 2012). Our previous study using a concordance index procedure showed that in the beginning of couple therapy (two spouses and two therapists), the couples showed the lowest level of EDA synchrony, client-therapist synchrony resembled that in individual psychotherapy, and the co-therapists showed the highest synchrony (Karvonen et al., 2016). Interestingly, the only change was observed among the couples, whose synchrony increased toward the end of therapy (Tourunen et al., 2020). This change was associated with a positive linear trend in the female clients’ wellbeing during the therapy process. Generally, heightened synchrony between participants was related to a better therapy outcome.

Synchrony between spouses has been studied in different laboratory tasks. However, the results may not be replicated in therapy situations. Whereas in a dyadic setting couples may show higher EDA synchrony during interactions involving negative affects (Levenson and Gottman, 1983; Coutinho et al., 2019), in our study of couple therapy we found lower synchrony in the beginning compared to the end of therapy, when the wellbeing of spouses improved (Tourunen et al., 2020). It is likely that the therapy situation itself and the work of therapists downregulates expressions of strong negative emotions and that this affects couples’ synchrony patterns. In a larger literature review, which also included studies of daily life, couples’ synchrony in physiological modalities was positively associated with relationship connectedness (cortisol was the only exception; Timmons et al., 2015). EDA synchrony might also reflect the emotional intensity of the interaction (Slovák et al., 2014) rather than specific positive or negative affects.

### Movement Synchrony

Synchrony in movement has often been measured using observer-based methods (e.g., Hall et al., 1995), although automatic computer algorithms, such as Motion Energy Analysis (MEA; Ramseyer and Tschacher, 2011; Ramseyer, 2020a), which was used in the present study, are increasingly being implemented. We have also developed and tested an observer-based body synchrony coding method for movement synchrony, which takes mimicry into account (Nyman-Salonen et al., 2021a). However, because of the differences between these types of analysis, our focus here is on MEA.

Movement synchrony between clients and therapists has been found especially in non-manualized therapies, but also in manualized therapies (Altmann et al., 2020). Movement synchrony has been suggested to be a process variable because of its effects on therapy outcome (Prinz et al., 2021). Ramseyer and Tschacher (2011) found that higher movement synchrony was related to symptom reduction and higher patient-rated relationship quality with the therapist. They also observed that body movement synchrony was associated with more immediate progress in sessions, whereas head synchrony was associated with the overall therapy outcome (Ramseyer and Tschacher, 2014). Low movement synchrony in the beginning phase of therapy has been associated with drop-out (Paulick et al., 2018a) and premature termination of therapy (Schoenherr et al., 2019).

However, the association between movement synchrony and therapy outcome is not always straightforward, and a high level of synchrony might not always be beneficial. Lower levels of movement synchrony at the beginning of therapy have been associated with fast improvements in interpersonal difficulties, and these patterns might relate to treatment outcome (Lutz et al., 2020). Movement synchrony might also be higher in sessions, where therapists evaluate lower progress (Ramseyer, 2020b). When therapists use supportive techniques, synchrony can be higher in the following therapy sessions (Deres-Cohen et al., 2021). Reasons for seeking therapy can also affect movement synchrony; for example, depressive patients may have lower synchrony than anxious patients with their therapists even when controlling for movement quantity (Paulick et al., 2018b). Attachment avoidance can result in a longer time lag until synchrony is established (Schoenherr et al., 2021).

We found that, like EDA synchrony in couple therapy (Tourunen et al., 2020), movement synchrony in couple therapy was associated with clients’ and therapists’ alliance evaluations and the therapy outcome (Nyman-Salonen et al., 2021b). For example, higher body movement synchrony during a session resulted in higher client-rated alliance at the end of the session, whereas therapists’ alliance ratings were related to both body and head synchrony. We also found gender differences in both EDA (Tourunen et al., 2020) and movement synchrony (Nyman-Salonen et al., 2021b) in how synchrony is associated with personal wellbeing and alliance.

### Speech

Speech synchrony was not a focus in this study; instead, the interest was in the relation between the physical action of talking and EDA and movement synchrony. On synchrony *per se*, the results have been conflicting. In one study, higher synchrony in vocal pitch was associated with higher empathy ratings (Imel et al., 2014), although this result was not replicated in a later study (Gaume et al., 2019). In another study, higher vocal pitch synchrony was associated with a poorer therapeutic relationship and greater distress (Reich et al., 2014).

In couple therapy the therapeutic dialogue is organized between multiple participants, and who talks and who listens changes constantly. Our focus was on the participants’ total amount of speech and amount of simultaneous speech. The latter was chosen as an additional measure as simultaneous speech has been given many interpretations, ranging from dominance and interruption (Ferguson, 1977) to “chiming in,” i.e., shared affective stance (Pfänder and Couper-Kuhlen, 2019). An interesting byproduct of this study is the information yielded about who talks in couple therapy. We are unaware of any studies that have reported the percentages of couple versus therapist talk in couple therapy. This is of course likely to depend on the style of the therapy as well as therapist-related factors. There is also emerging evidence of synchrony in neural activity and psychophysiological processes during conversations (Finset, 2014). The question remains how synchrony in different modalities are connected to each other.

### Connecting the Different Modalities

To our knowledge, no studies exist on whether ANS synchrony and movement synchrony are connected in psychotherapeutic settings. Moreover, no studies have investigated how the motor action of speaking affects these synchrony modalities. In individual psychotherapeutic settings, coordination in movement has been associated with therapy outcome, coordination in physiology with empathy, and coordination in other modalities (such as language) with the therapeutic alliance (Wiltshire et al., 2020). The fact that talking can increase the level of arousal and amount of movement prompts an important question of whether non-verbal synchrony is only a byproduct of vocal communication patterns or something more.

The relationship between ANS and behavioral synchrony is unclear (Palumbo et al., 2017; Mayo and Gordon, 2020), but it seems reasonable to assume that since EDA generally rises in experiencing emotions (Kreibig, 2010), and emotions are thought to reflect preparation for motor action (Fredrikson et al., 1998; Brehm, 1999), SNS synchrony, movement synchrony and speech can be associated with each other. Movement synchrony may be more strongly associated with speech than with EDA synchrony, as talking is often accompanied by head and body movements, which cause pixel changes in the videos used in MEA analyses. In couple therapy, we observed that movements, especially of the head, were usually speech-related, signaling turn-taking or including nodding while talking or listening (Nyman-Salonen et al., 2021b).

Our previous research has focused on single modalities of synchrony and how they are related to alliance and outcome. Here our interest was to study the associations between different synchrony modalities and speech.

The research questions were as follows:

1. Are EDA synchrony, movement synchrony, and speech (total amount and amount of simultaneous speech) associated with each other in couple therapy?
2. Do the relative amounts of EDA synchrony, movement synchrony, and speech change from the beginning to the end of therapy?

## Materials and Methods

### Data

Couple therapy was implemented in the Psychotherapy Training and Research Center, University of Jyväskylä, Finland. The data analyzed in this study were from the “Relational Mind in Events of Change in Multiactor Therapeutic Dialogues” research project (Seikkula et al., 2015). Details on participants and procedures and on theoretical aspects of interpersonal synchrony are presented in Karvonen (2017).

Briefly, the data comprised 12 couple therapy processes: 24 clients and 10 different therapists, who worked in pairs with each couple. Thus, four people in six different dyad combinations were present in each therapy session. The clients were on average 43 years old (range 28–61), and the therapists 52 years old (range 32–63) at the time of the study. Eleven couples comprised a female and a male client, and one couple comprised two female clients. Nine of the co-therapist dyads were opposite-sex and three same-sex dyads.

The therapy sessions were scheduled to last 90 min. The four participants were seated equidistantly in a circular arrangement around a center table, the clients next to each other and the therapists next to each other. The therapists worked in their habitual style, mostly either dialogical or narrative, and usually engaged in reflective discussions between themselves toward the end of the therapy sessions. All sessions were recorded with six high quality video cameras: one camera for capturing each participant’s precise facial image, and two cameras to capture whole body images of both clients and therapists (one camera filming clients and one filming therapists).

Participants’ autonomic nervous system activity during the therapy sessions was measured twice from the same therapy case: in the initial phase of couple therapy, and toward the end of the therapy. The first recording was made during the second therapy session (in one case the third, owing to scheduling problems), and the second in session five, six, or seven (*Mdn* = 6), depending on the time the therapy was estimated to last. The same therapist dyad worked with the couple throughout therapy.

Nine therapies continued long enough to have a second ANS recording in or near to the sixth therapy session. In three therapy cases, the clients and the therapists decided together that the therapy process can end before the fifth session, so the participants were not asked to schedule an extra session just for research purposes. One end-of-therapy session was excluded from the analysis, as the couple had brought their baby to the session, which negatively affected the quality of the movement data. The final data thus consisted of 72 dyads for the analysis of EDA and movement synchrony and speech at the beginning of therapy (12 couple therapy sessions), and 48 dyads for the same measurements toward the end of therapy (eight couple therapy sessions) (Table 1). Note that here “a dyad” represents any two participant combination of the couple therapy session (four different client-therapist dyads, one client-client dyad, and one therapist-therapist dyad), compared to previous literature about individual psychotherapy, in which a dyad represents one client and one therapist.

**TABLE 1:** Descriptive statistics of the studied variables.

	Beginning of therapy					End of therapy				
	*n*	Mean	SD	Min	Max	*n*	Mean	SD	Min	Max
**EDA synchrony**	72	3.529	7.476	−12.836	43.416	48	2.965	4.435	−4.192	16.146
Couple	12	0.461	7.232	−12.836	14.972	8	1.774	2.826	−4.192	4.840
Client-therapist	48	3.079	5.341	−5.776	19.683	32	2.225	3.738	−2.820	12.489
Co-therapists	12	8.394	12.232	−1.705	43.416	8	7.116	6.195	−1.144	16.146
**MEA body synchrony**	72	−0.104	4.175	−12.938	10.305	48	0.079	3.467	−7.868	7.419
Couple	12	−1.802	3.845	−12.938	1.044	8	0.918	2.501	−3.253	4.103
Client-therapist	48	−0.419	4.136	−9.025	10.305	32	−0.756	3.381	−7.868	7.419
Co-therapists	12	2.850	3.396	−4.524	8.040	8	2.579	3.566	−2.463	7.339
**MEA head synchrony**	72	2.583	7.031	−5.987	39.652	48	1.736	4.779	−13.050	14.548
Couple	12	−1.428	2.375	−5.987	1.306	8	−0.908	5.740	−13.050	7.438
Client-therapist	48	2.782	7.796	−5.612	39.652	32	1.029	3.451	−7.275	7.810
Co-therapists	12	5.795	4.975	1.848	20.300	8	7.207	4.814	0.421	14.548
**Total speech**	72	0.506	0.176	0.136	0.923	48	0.480	0.170	0.161	0.866
Couple	12	0.752	0.110	0.585	0.923	8	0.668	0.180	0.339	0.866
Client-therapist	48	0.506	0.107	0.259	0.707	32	0.480	0.124	0.255	0.734
Co-therapists	12	0.261	0.087	0.136	0.419	8	0.291	0.115	0.160	0.488
**Simultaneous speech**	72	0.023	0.022	0.0004	0.0996	48	0.022	0.022	0.001	0.090
Couple	12	0.046	0.031	0.012	0.100	8	0.037	0.025	0.004	0.086
Client-therapist	48	0.021	0.018	0.0004	0.0587	32	0.020	0.022	0.0006	0.0904
Co-therapists	12	0.010	0.010	0.001	0.034	8	0.012	0.012	0.002	0.033

*Synchrony values are expressed as SUSY ES_noabs_ scores and the speech values represent the percentages of time each dyad talked during the coupe therapy session.*

### Electrodermal Activity

The electrodermal activity of each therapy participant was recorded with two skin conductance electrodes (Ag/AgCl, Ambu^®^ Neuroline 710, Ballerup, Denmark) from the participant’s non-dominant palm, below the first and fourth digits. An amplifier (Brain Products Brainamp ExG 16, Brain Products, Gilching, Germany) and data acquisition program (BrainVision Recorder, Brain Products, Gilching, Germany) were used to record EDA with a sampling frequency of 1,000 Hz. Skin conductance was determined with a constant voltage of 0.5 V (GSR sensor, Brain Products, Gilching, Germany), the signal was amplified in the DC mode and low-pass filtered at 250 Hz. The recorded data was downsampled offline to 10 Hz using a Brain Vision Analyzer (Brain Products, Gilching, Germany) and written to a text file for further analyses.

We have previously used the same electrodermal data in Tourunen et al. (2020) (except that in this study we omitted the session with the baby in it), but the preprocessing of the data differed. In the present study we did not preprocess the signal and thus both skin conductance levels and skin conductance responses were included in the EDA signal. In our previous studies (Karvonen et al., 2016; Tourunen et al., 2020) sample to sample differences were calculated to detect change. The concordance index thus determined the synchrony of skin conductance responses.

### Motion Energy Analysis

Videos of the couple therapy sessions were analyzed using MEA, an automated video-analysis algorithm designed to quantify movements (Ramseyer and Tschacher, 2008). Motion energy is defined as the amount of gray-scale pixel changes occurring between consecutive video-frames. The calculation is made within a manually defined region of interest (ROI).

For this study, the head and the body of each therapy participant was selected separately, resulting in eight ROIs. These were checked manually in each video before data extraction in order to exclude overlapping movement between the different ROIs. After this, using an extraction precision of 10 fps (as in the videos), the MEA generated a time series of pixel changes for each ROI. To exclude video noise, the threshold for recording pixel changes was the preprocessing default (15). In addition, any spurious peaks at the beginning of the recording (duration less than 1 s) were removed.

A specially made marker unit was used to synchronize the ANS measures to the therapy videos, giving a simultaneous transistor-transistor logic (TTL) pulse (ANS) and sound (video), to which the data was edited to begin with.

We have previously analyzed movement synchrony from the same data using 10 Hz time series for the SUSY computations. The previous study used a larger data set, which contained measurement sessions (*N* = 17) as well as regular sessions (*N* = 12) in which electrodermal activity was not recorded (Nyman-Salonen et al., 2021b).

### Speech

Coding of speech was done by psychology students, who were given training for the task. Usually, one student coded ca. three therapy sessions, and coding of the same session was not divided between students. Speech in the therapy session videos was manually coded separately for each person per every second: 0 = not talking, 1 = talking. Coders first located all speech turns for one participant by marking time stamps for the start and end of a speech turn to an Excel sheet. This included single words, such as “yes.” A speech turn was analyzed more thoroughly and sectioned in parts, if a silence over 1 s was observed inside the speech turn. After that, time series for the sessions were prepared by transferring the values (value 0 or 1 for each second for each participant) to another Excel sheet.

A dyadic variable, *Total speech*, was calculated from the sum total of seconds of speech (number of 1 s) for couples, the client-therapist dyads and co-therapists. The dyadic variable *Simultaneous speech* was calculated as the number of seconds both individuals were talking at the same time (both assigned the value 1 for that second). In the case of overlapping speech in the variable *Total speech*, the dyad was assigned the value 1 if either or both individuals were speaking during that second.

Because the therapy sessions varied in duration, the number of seconds of each participants’ speech was divided by session duration to obtain comparable percentage values for all participants. For example, in the beginning of therapy, the mean total speech of couples accounted for 75% (0.752) of the session time (Table 1).

### Computing Synchrony

Synchrony has been calculated using several different procedures. EDA synchrony has been calculated using a concordance index procedure (Marci and Orr, 2006; Karvonen et al., 2016; Tourunen et al., 2020), as well as windowed cross-lagged correlations (the Surrogate Synchrony procedure) (Coutinho et al., 2019; Tschacher and Meier, 2020). Movement synchrony in psychotherapy has mainly been calculated using windowed cross-correlations such as the SUSY procedure (Ramseyer and Tschacher, 2011, 2014), but also windowed cross-lagged regression has been used (Altmann et al., 2021), as well as cross-recurrence quantification analysis (cf. Schoenherr et al., 2019). For a detailed comparison between the different synchrony calculations see Delaherche et al. (2012) and Schoenherr et al. (2019).

We have previously used the concordance index procedure to study synchrony in EDA (Karvonen et al., 2016) and its relation to the alliance and outcome (Tourunen et al., 2020), and the Surrogate Synchrony procedure for calculating movement synchrony (Nyman-Salonen et al., 2021b).

### Surrogate Synchrony Procedure for Electrodermal Activity and Motion Energy Analysis

In the present study, we used the same synchrony computation algorithm for EDA and movement to obtain comparable synchrony values between the two modalities. Synchrony was computed using the Surrogate Synchrony (SUSY) procedure (Tschacher and Haken, 2019, 2020) in a web-based app^1^. SUSY computes dyadic synchrony, hence six dyads in each therapy session were analyzed: client 1 – client 2, client 1 – therapist 1, client 1 – therapist 2, client 2 – therapist 1, client 2 – therapist 2, and therapist 1 – therapist 2. The MEA and EDA time series were first downsampled to 1 Hz for comparability (by averaging 10 successive samples) and then divided into 30-s segments, in which cross-correlations were calculated using time lags of ±5 s by shifting one time series in relation to the other in 1-s steps (1 Hz sampling rate). This time lag was chosen, because it has been used in movement synchrony computations, and it was still compatible with our previous research on EDA synchrony in which we used a ±7 s lag. Cross-correlations were standardized using Fisher’s *Z* and then aggregated to a mean *Z* value of synchrony separately for all lags in each segment. The mean *Z* values of all segments were averaged, resulting in a mean *Z* value of non-verbal synchrony for the whole therapy session for each dyad and synchrony type (EDA synchrony, MEA body synchrony, and MEA head synchrony).

Surrogate synchrony calculates both absolute (*Z*_abs_) and non-absolute (*Z*_noabs_) mean *Z* synchrony values from the cross-correlations. *Z*_abs_ are thus calculated by converting negative values into positive ones, and *Z*_noabs_ by using the original positive and negative values of the cross-correlations. Non-verbal synchrony tends to change during interaction, so it is often recommended to use statistical models that account for these fluctuations (Palumbo et al., 2017), thus taking into account both in-phase (positive cross-correlations) and anti-phase (negative cross-correlations) synchrony (Tschacher and Meier, 2020).

In order to confirm that the calculated synchrony does not occur merely by chance, segments of the original time series were shuffled to create “pseudo synchronies” to estimate whether synchrony occurred above chance level. Successive EDA and MEA values are also not independent of each other, so it is important to account for autocorrelation. These pseudo synchronies were then computed in the same way as the actual synchrony computations, with an upper limit on surrogates of *n* = 1,000. Empirical synchronies were standardized using the mean value of pseudo synchronies, resulting in both absolute (ES_abs_) and non-absolute value effect sizes (ES_noabs_) for EDA synchrony, MEA body synchrony, and MEA head synchrony. We used the non-absolute value effect size (ES_noabs_), because it enables in-phase synchrony to be distinguished from anti-phase synchrony, and since it was used in Nyman-Salonen et al. (2021b). The non-absolute value effect size (ES_noabs_) is more comparable to the concordance index procedure we have used with EDA (Karvonen et al., 2016; Tourunen et al., 2020). The formula to calculate the non-absolute effect size was: ES_noabs_ = (*Z*_noabs_ – Pseudo *Z*_noabs_)/SD(Pseudo *Z*_noabs_). These ES_noabs_ effect sizes were used in all analyses.

To calculate whether the synchrony values between each of the six dyads were significant in each session, the significances for the effect sizes (ES_noabs_) of each dyadic synchrony value for both EDA and MEA (head and body movement) per session were computed using one-sample *t*-tests.

### Statistical Analyses

Due to the nested data (two couple therapy sessions in each therapy case), associations between the different synchrony modalities and speech were analyzed using one-level models with complex method in Mplus v8.4 [Similarly, as was done in Nyman-Salonen et al. (2021b)]. For each therapy case, synchrony analyses were conducted for six dyad combinations, each of which was used as a different variable. Two-level intraclass correlations (ICCs) were computed for within (level 1) and between (level 2) therapy cases to account for the fraction of the total variation in the data that is accounted for by between-group variation. Table 2 shows the ICCs for EDA and movement synchrony. Between-group variation was prominent for couples’ body movement synchrony, all the client-therapist EDA and movement synchronies, and co-therapist EDA synchrony. Hence, complex method was needed in order to take into account hierarchical data. Two-level models were not used because the main interest was not in the difference between within and between levels.

**TABLE 2:** Intra-class correlations of synchrony modalities.

	Intra-class correlations			
	ICC	SE	Est. SE	*p*
**EDA synchrony**				
Couple	0.100	0.195	0.510	0.610
Client-therapist	0.770	0.133	5.794	<0.001
Co-therapists	0.339	0.111	3.058	0.002
**MEA body synchrony**				
Couple	0.199	0.061	3.279	0.001
Client-therapist	0.666	0.115	5.780	<0.001
Co-therapists	0.141	0.273	0.517	0.605
**MEA head synchrony**				
Couple	0.011	0.139	0.079	0.937
Client-therapist	0.264	0.064	4.115	<0.001
Co-therapists	0.017	1.199	0.014	0.989

Models were computed using Full Information Maximum Likelihood (FIML) estimation and maximum likelihood with robust standard error (MLR) estimator (values missing at random). FIML is an efficient estimator for models that are non-linear in their parameters, and MLR makes the results robust to non-normality and non-independence of observations. MLR standard errors were calculated using a sandwich estimator, which takes nested data into account by correcting the standard errors, thus producing *p*-values that are more reliable.

To compare changes in synchrony and speech from the beginning of therapy to the end of therapy, paired samples *t*-tests were calculated using SPSS v26. Differences between the measuring points were studied first for the whole sample and then for the subgroups (couples, client-therapist dyads, and co-therapists). Because there were six dyad synchrony values per therapy session and four of these were dyad synchrony values between clients and therapists, means were calculated for the whole sample and client-therapist dyads before analyses to account for interdependence of observations. As sessions were treated as separate variables in these analyses, observations, that is 8 therapy cases, were independent of each other and thus not hierarchical. Because sessions were treated as separate variables in these analyses, observations, that is 8 therapy cases, were independent of each other and thus not hierarchical. Effect sizes for within-group comparisons were calculated using paired samples Cohen’s *d*.

## Results

Both in-phase (positive) and anti-phase (negative) synchrony were found in EDA and movement (MEA) (Table 1). The majority of the synchrony values were statistically significant on a *p*-level of <0.05: EDA synchrony (118/120), movement synchrony (232/240). On average, participants’ synchrony tended to be in-phase throughout the therapy, although more anti-phase body synchrony was observed at the beginning of therapy (ES_noabs_ = −0.10). However, as shown by the standard deviations and minimum and maximum values, the effect sizes varied widely. The co-therapists’ head synchrony was the only synchrony that was invariably in-phase during therapy. On average, the client-therapist body synchrony and couples’ head synchrony were anti-phase throughout therapy.

On average, the couple talked for 67–75% and the co-therapists for 26–29% of the session time. The percentage of simultaneous speech in the therapy sessions was 4–5% among couples, 2% between clients and therapists, and 1% between co-therapists.

### Associations Between Synchrony Modalities and Speech

Table 3 shows the correlations between the synchrony modalities and speech variables for all participants and each subgroup, i.e., couples, client-therapist dyads, and co-therapists. In general, across the different dyads, EDA synchrony correlated strongly with movement synchrony (*r* = 0.81 body, *r* = 0.72 head), but not with the amount of speech or simultaneous speech. Body and head synchrony were associated (*r* = 0.75), and both correlated negatively with the amount of simultaneous speech in the dyads (*r* = −0.53 body, *r* = −0.37 head).

**TABLE 3:** Estimated correlations between synchrony modalities and speech using the complex method.

	MEA body synchrony	MEA head synchrony	Total speech	Simultaneous speech	Mean	Model estimate *p*
**EDA synchrony**	0.808**	0.721**	−0.260	−0.288	3.303	<0.001
Couple	0.246	0.156	−0.036	−0.137	0.986	0.466
Client-therapist	0.663**	0.590**	0.022	−0.449*	2.737	<0.001
Co-therapists	0.092	0.128	−0.300	−0.024	7.883	0.003
**MEA body synchrony**		0.751**	−0.370	−0.532**	−0.031	0.961
Couple		0.285	−0.623**	−0.274	−0.714	0.424
Client-therapist		0.766**	−0.228	−0.458*	−0.553	0.495
Co-therapists		0.665**	−0.385**	−0.369	2.742	0.001
**MEA head synchrony**			−0.164	−0.368*	2.244	0.025
Couple			−0.470**	−0.192	−1.220	0.139
Client-therapist			0.094	−0.292	2.081	0.121
Co-therapists			−0.281**	−0.169	6.360	<0.001

*p < 0.05, **p < 0.01, N = 20 sessions.

**FIGURE 1:**
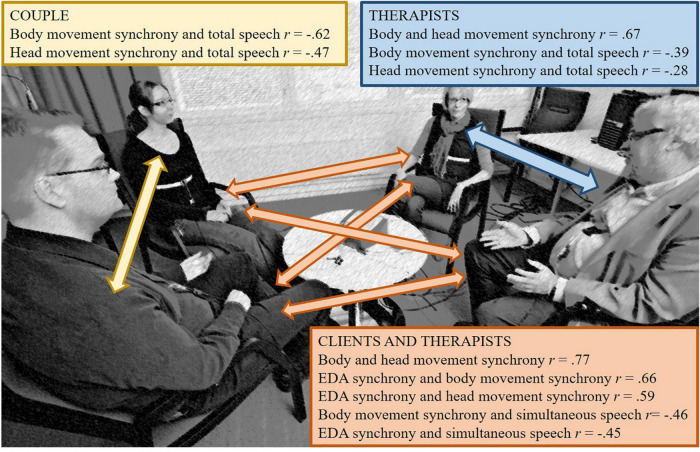
Simulation picture of the research setting (no actual clients) with arrows representing dyad combinations, and a summary of all statistically significant correlations between synchrony modalities and speech.

Figure 1 illustrates all the statistically significant correlations between the different synchrony modalities and speech in each subgroup. Movement synchrony had a negative correlation with the amount of speech during the therapy sessions among couples and co-therapists, meaning that the dyads had more movement synchrony when they talked less (and vice versa). EDA and movement synchrony walked “hand in hand” only between clients and therapists. These two synchrony measures also correlated negatively with the amount of simultaneous speech in the therapy sessions, indicating less synchrony when clients and therapists talked on top of each other.

### Changes From the Beginning of Therapy to the End of Therapy

Table 1 shows the descriptive statistics of all studied variables at the beginning and end of therapy. No statistically significant differences between the synchrony modalities and speech were observed between the two measurement points based on a p-level of <0.05. Cohen’s *d* produced small (>0.2) and close to large (0.8) effects, which are described below:

The effect sizes indicated that more anti-phase body synchrony between participants occurred across the whole sample at the beginning of therapy (*M* = −0.48, SD = 2.76) than at the end of therapy [*M* = 0.08, SD = 2.06; *t*(7) = −0.77, *p* = 0.469, Cohen’s *d* = 0.23]. However, the participants showed more in-phase head synchrony at the beginning of therapy (*M* = 2.72, SD = 5.69) than at the end of therapy [*M* = 1.74, SD = 2.54; *t*(7) = 0.54, *p* = 0.605, Cohen’s *d* = 0.22].

Concerning subgroups, couples talked more at the beginning of therapy (*M* = 0.72, SD = 0.13) than at the end of therapy [*M* = 0.67, SD = 0.18; *t*(7) = 1.67, *p* = 0.139, Cohen’s *d* = 0.36]. There was also less simultaneous speech at the end of therapy [beginning of therapy *M* = 0.05, SD = 0.04; end of therapy *M* = 0.04, SD = 0.02; *t*(7) = 1.67, *p* = 0.310, Cohen’s *d* = 0.36]. Clients and therapists showed more head synchrony at the beginning of therapy (*M* = 3.34, SD = 7.80) than at the end of therapy [*M* = 1.03, SD = 2.85; *t*(7) = 0.99, *p* = 0.356, Cohen’s *d* = 0.39]. Co-therapists showed less head synchrony at the beginning of therapy (*M* = 4.43, SD = 2.06) than at the end of therapy [*M* = 7.21, SD = 4.81; *t*(8) = −1.42, *p* = 0.199, Cohen’s *d* = 0.75]. They also talked more toward the end of therapy [beginning of therapy *M* = 0.25, SD = 0.10; end of therapy *M* = 0.29, SD = 0.11; *t*(7) = −2.24, *p* = 0.06, Cohen’s *d* = 0.40], and produced more simultaneous speech at the end of therapy [beginning of therapy *M* = 0.01, SD = 0.01; end of therapy *M* = 0.01, SD = 0.01; *t*(7) = −0.84, *p* = 0.06, Cohen’s *d* = 0.24].

## Discussion

The main aim of this study was to assess whether different modalities of interpersonal synchrony are associated with each other in couple therapy. We were especially interested in comparing involuntary synchrony in the SNS to more voluntary synchrony in the body and head movements of therapy participants. Further, we wanted to examine these synchronies in relation to the physical act of talking, in order to evaluate whether non-verbal synchrony was related to the amount of verbal communication in therapy sessions. To our knowledge, the association between SNS synchrony, movement synchrony and speech has not been studied earlier in couple therapy. In general, we found strong positive correlations between SNS synchrony and movement synchrony, and negative correlations between simultaneous speech and movement synchrony (moderate for body synchrony, weak for head synchrony) when looking at all the possible dyadic combinations. However, the subgroup analyses revealed differences in these associations between the couples, client-therapist dyads, and co-therapists. These differences should be taken into account when interpreting the results.

Despite the wide variation between the dyads in their level of synchrony, including both in-phase and anti-phase synchrony, on average the couple therapy participants’ synchrony occurred in-phase. Anti-phase synchrony was more evident in movement than in EDA, especially within couples and between clients and therapists. This might be related to the dynamics of turn-taking in a multiactor setting, such as in-phase movement synchrony reflecting mutual and simultaneous involvement and anti-phase movement synchrony reflecting giving space to others (one person talking and the other listening) (Nyman-Salonen et al., 2021b).

In-phase and anti-phase synchrony may also serve distinct social functions (Miles et al., 2010). However, it is important to note that the term “anti-phase” movement synchrony has been used to describe both morphological (behavioral mirroring or matching) and temporal synchrony. Morphological synchrony typically involves counter positioning such as one person being in full extension while the other is in full flexion (Miles et al., 2010), or one person having their hand up while the other’s hand is down (Macrae et al., 2008). In the present study, anti-phase synchrony refers to movement energy in opposing directions, that is, negative synchrony during which one participant starts to move more while the other starts to move less (Tschacher and Meier, 2020; Coutinho et al., 2021; Nyman-Salonen et al., 2021b). Temporal movement synchrony can have different associations with phenomena like empathy, rapport, and anxiety, compared with morphological movement synchrony (Bernieri, 1988; Fujiwara and Daibo, 2021).

So far, studies have not really offered an interpretation of temporal anti-phase synchrony. In this study, as in our previous study (Nyman-Salonen et al., 2021b), the co-therapists’ body and head movement synchronies were in-phase, most likely indicating that they were observing and reacting to external events at the same time (Butler, 2015; Tourunen et al., 2020). An interesting finding was that only movement synchrony and speech, not sympathetic nervous system synchrony, seemed to change during the course of therapy. At the beginning there was more in-phase head synchrony and anti-phase body synchrony between all participants than at the end of therapy. As movement synchrony is more voluntary than SNS synchrony, our finding could reflect changes in how the participants worked together during the course of therapy, which is compatible with clinical observations. Previous research on movement synchrony during individual therapy processes has not yielded similar results (Ramseyer, 2020b).

An important finding for future research was that EDA and movement synchrony were associated with each other only in the client-therapist dyads (*r* = 0.66 for body synchrony and *r* = 0.59 for head synchrony). The fact that a similar connection was not found between spouses or therapists who worked together might testify to the special role of the therapeutic alliance (Koole and Tschacher, 2016; Tourunen et al., 2020; Nyman-Salonen et al., 2021b) and empathy (Marci and Orr, 2006; Marci et al., 2007; Messina et al., 2013) in therapeutic interactions. Future research should aim to clarify whether non-verbal synchrony has a special association with the “bond” aspect of the therapeutic alliance (Bordin, 1979), which focuses on client-perceived trust, respect, and caring in therapy (Johnson and Wright, 2002). Coordination in movement and speech could underpin the therapeutic relationship, whereas coordination in physiology could capture the “real” emotional relationship (Wiltshire et al., 2020). The more clients and therapists spoke on top of each other, the less EDA and movement synchrony they showed, suggesting that a good flow of interaction may also be important for synchrony.

The assumption that movement synchrony would show a stronger association with speech than EDA synchrony was supported. However, contrary to expectations, talking actually correlated negatively with all the synchrony measures. We thought that the more the members of a dyad talked during the therapy session, the more synchrony they would exhibit in movement and arousal. However, the couples and co-therapists showed less in-phase movement synchrony in sessions where they spoke more (and vice versa). Non-verbal behavior may preface speaking turns (Harrigan et al., 1985), and cultural and language style matching may inversely correlate with body movement (Dale et al., 2020). Furthermore, talking requires retrieving different kinds of information from memory and combining them into larger structures (Hagoort and Levelt, 2009). These processes can disrupt bodily attunement by increasing cognitive load and directing attention toward the self. Speaking also involves non-verbal movements and gestures, which reflect self-oriented cognitive functions in addition to communicative functions (Lausberg and Kita, 2003; Kita et al., 2017).

An interesting topic for future research is whether synchrony and speech can work as complementary processes in which a connection between persons is established through either non-verbal synchrony or words. Movement synchrony and language style matching may reflect a trade-off, whereby one modality of synchrony is present, the other need not be (Dale et al., 2020). In a case study involving the precise analysis of a 6-min couple therapy segment, we found that participants who were not involved in the conversation displayed synchrony in several different modalities during moments which required non-verbal support or repair of the connection (Kykyri et al., 2019). Verbal abstinence by therapists can support therapeutic work by keeping difficult feelings activated while resonant non-verbal behavior enhances the working alliance (Bänninger-Huber and Widmer, 1999).

The findings on the division of speech in couple therapy were as expected. The couples talked more (∼71% of session time) than the therapists (∼28% of session time). This likely reflects couple therapy in general, although there may be differences depending on the therapeutic approach used, the therapists themselves and cultural factors. However, few studies have addressed cultural differences in relation to interpersonal synchrony. For example, in Finland generally, silence is well tolerated and personal space is important, whereas in France verbal and non-verbal rapport may be more important (Isosävi, 2020). Compared to the beginning of therapy, the couples seemed to talk less and produced less simultaneous speech toward the end of therapy, whereas the therapists appeared to talk more and produced more simultaneous speech. Intuitively, this result seems reasonable, since in the beginning of therapy therapists require a lot of information about the clients and their past.

It is important to note that the coding of speech in seconds did not contain information on to whom the talk was addressed. That is, the total speech produced by a dyad was not a measure of how much of the talk by the two individuals was directed to each other or to others, but simply the amount of time spent speaking aloud. Limiting the coding of who speaks or does not speak is a straightforward approach that does not, of course, capture the intricacy of therapeutic conversations. Instead of quantitative dominance in speech, interactional and semantic dominance may be more important for dialogue in therapy (Seikkula and Olson, 2016). Moreover, clients and therapists have many ways to actively participate in the therapeutic process other than speaking aloud.

### Limitations and Methodological Considerations

One limitation in this study is the sample size of twelve couple therapy cases. Although 48–72 dyads is adequate for overall statistical testing using complex modeling for nested data, the subgroup analyses pointed to the need of assessing the couple, client-therapist and co-therapist dyads separately, which reduces statistical power. Especially the findings concerning changes from the beginning of therapy to the end of therapy should be regarded as exploratory because of the small number of therapy cases (8). When interpreting the results of the analysis, it should be remembered that the client-therapist dyad values are the means of four dyad combinations in each session. One difficulty with the data in multiactor settings is the use of the same participant’s data in different dyadic synchrony computations. In future studies there is a need to find more sophisticated methods to study synchrony between several participants.

Another issue is the large variance in synchrony scores. Together with the small sample size, it is hard to obtain statistically significant results using tests that rely on normal probability distributions. Although the paired samples *t*-test *p*-values fell below the 0.05 threshold for detecting changes in the studied variables, we reported Cohen’s *d* effect sizes for all the findings that might be interesting for future research. The current scientific consensus supports the use of effect sizes rather than relying solely on *p*-values (e.g., Sullivan and Feinn, 2012). Sample size and correlations between measurement points (such as the different therapy sessions treating the same case) affect *p*-values, whereas Cohen’s *d* uses means and standard deviations.

Previously, using a concordance index procedure on the same data, we found that EDA synchrony occurred between the majority of the dyads and increased between the spouses from the beginning to the end of therapy (Tourunen et al., 2020). However, in this study the windowed cross-correlation calculated for the same data with the SUSY procedure did not yield a similar result concerning the change from beginning to end. The explanation may lie in the greater variance in the SUSY effect sizes than concordance indices and thus a lack of statistical power in detecting change, since the descriptive statistics indicated a similar change. Another possibility is that the skin conductance responses analyzed using the concordance index procedure may be more sensitive for detecting change over sessions than the combined skin conductance levels and responses analyzed using the SUSY procedure. Time lags in the calculations were also slightly different in the two approaches (concordance index 15 s versus SUSY 10 s). Moreover, it should be noted that there were more significant EDA synchronies when using the SUSY procedure compared to the concordance index procedure. Albeit, we chose to calculate synchrony using the SUSY procedure, because of its extensive use in psychotherapy studies.

Considering our previous study on movement synchrony where we used the SUSY procedure with a larger data set, including also non-measurement, e.g., regular sessions, the results were similar to the present study (Nyman-Salonen et al., 2021b). However, according to our previous study regular and measurement sessions differed in the amount of head synchrony between the co-therapists, which should be taken into account when interpreting the results of the present study.

In general, the different methods used to analyze synchrony tend to produce different results (Palumbo et al., 2017; Tschacher and Meier, 2020), since they do not measure the same aspects of synchrony (Schoenherr et al., 2019), and since they compute synchrony in various ways. The choice of parameters also affects synchrony values. For example, a window size of 60 s has been common in previous research for analyzing movement synchrony in 15-min segments, but our tests of different parameters and the quality of the analyses gave the best differentiation between real and pseudosynchrony values using a shorter 30-s window for the 90-min therapy sessions.

Until now a major focus has been on whether synchrony occurs above chance level. There is a need to take a further step for understanding different aspects of synchrony. By using the SUSY procedure and especially the in-phase and anti-phase synchrony values, we wanted to separate between the different kinds of synchronies, which occur during interactions.

For extracting the participants’ movements we used MEA as an automatic movement tracking technique. The advantages of video-based automatic movement tracking techniques, such as MEA or OpenPose (Cao et al., 2017) are in simplicity and accessibility (Fujiwara and Yokomitsu, 2021). However, compared to more time-consuming observer-based methods, they lack information about the direction and form of movement. In the MEA analysis, one person nodding “yes” and the other person shaking their head “no” can appear as synchrony. Thus, researchers need to make decisions about what they want to focus on, for example based on underlying theories or research questions. Information is currently lacking on how results of temporal analyses compare with morphological analyses of movement synchrony in psychotherapeutic settings.

In the future, it would be important to do dynamic and system analyses that also account for triadic and quadratic relationships in couple and family therapy. Some examples of these include round-robin network approach, Granger causality, recurrence quantification models, complexity models (e.g., sample entropy), mutual information models as well as other advances in dynamic time series network analyses. These approaches allow dynamic interaction across modalities, and robustness in relation to non-stationary and non-parametric data.

Finally, it should be noted that focusing on session level phenomena and aggregated data is a starting point in understanding the associations between different synchrony modalities in therapeutic settings. Mixed method approaches and micro-level process analyses are likely to shed more light on how people attune with each other verbally and non-verbally (Kykyri et al., 2019). There may be certain aspects of therapy, that are more relevant for synchrony research than others, for example connecting information about synchrony with ruptures (Deres-Cohen et al., 2021), attachment security (Palmieri et al., 2018) or in couple therapy, patterns of “soothing” or “spiraling” (Gottman et al., 1998).

## Conclusion

Couple therapy provides an interesting setting not only for study of the therapeutic relationship between clients and therapists, but also between romantic partners and colleagues who work together. It is also a good setting for studying the unfolding of interpersonal synchrony in a natural flow of interaction, without laboratory tasks or restricting the conversation to specific topics. Nevertheless, studying multiple individuals entails complexity.

Our findings on the client-therapist relationship support the idea that ANS and movement synchrony are associated (Zhang et al., 2016), whereas our findings on couples and co-therapists suggest that they may also be independent processes (Codrons et al., 2014; Noy et al., 2015). The task of a couple clearly differs that of the therapists, and despite therapists’ best efforts, equality in the power structure is unlikely to be realized. Several alliances are established and maintained simultaneously, the participants observe the alliances of others, and hidden agendas may influence how therapists create and balance alliances (Friedlander et al., 2006a,b). Thus, owing to the interactional patterns and the fact that synchrony may serve different purposes in these relationships, it was hardly surprising that that the associations between the synchrony modalities varied among the dyad subgroups.

Research on interpersonal synchrony varies widely in several respects, including terminology, study designs and methodology. Synchrony may be more dependent on social context than the type of relationship or specific affects, and it can develop through a number of different mechanisms – including a shared environment and responses to a third variable (Palumbo et al., 2017). Therapy sessions are in many ways unique social situations, hindering generalizations from studies conducted in different settings. Understanding the functions and purpose of interpersonal synchrony seems clinically important, because research has shown that synchrony is associated with the therapeutic relationship and therapy outcome (Mende and Schmidt, 2021). Results of this study support the idea that there might be something unique the client-therapist relationship compared to romantic or collegial relationships, because automatic reactions in the nervous system and body movements acted in unison only in this therapeutic relationship. In practice, our results also point out the need to assess the physical act of speaking when studying synchrony, because the amount of non-verbal synchrony and the amount of talking had an inverse relationship.

The relationship between physiological and behavioral interpersonal synchrony is poorly understood, and thus calls for much more research (Mayo and Gordon, 2020). Our findings suggest that SNS synchrony between therapy participants does not reflect motor actions of gestures or speech alone. SNS synchrony may also be present in the absence of observable action, for example in emotional and cognitive processes. To understand more about the functions of interpersonal synchrony in therapy means considering the fluctuating aspects of interaction and participants’ possible goals at specific moments. Having more synchrony with others is not always beneficial: for example, too much synchrony can be detrimental for self-regulation (Galbusera et al., 2019), reinforce clients’ disadvantageous interpersonal patterns (Lutz et al., 2020) or indicate high levels of emotional contagion or transference (Bänninger-Huber and Widmer, 1999). Constant high in-phase synchrony with others all the time would quickly lead to dysfunction in interaction and prevent the performance of complex tasks (Dale et al., 2020). To better understand the complex patterns of interpersonal attunement requires more information on the links between different synchrony modalities (Scheidt et al., 2021).

## Data Availability Statement

The datasets presented in this article are not readily available because data set deals with highly sensitive and personal material (therapy videos etc.), which cannot be shared. Requests to access the datasets should be directed to V-LK, virpi-liisa.kykyri@jyu.fi.

## Ethics Statement

The studies involving human participants were reviewed and approved by the Human Sciences Ethics Committee, University of Jyväskylä, Finland. The patients/participants provided their written informed consent to participate in this study. Written informed consent was obtained from the individual(s) for the publication of any potentially identifiable images or data included in this article.

## Author Contributions

AT, JS, MP, and V-LK involved in designing the original research and collecting the data. AT performed the statistical analyses and were the main author of the research manuscript. PN-S performed the MEA extractions and provided the content for the movement analyses and interpretations. JM performed the Mplus statistical analyses. V-LK supervised the research assistants who worked on the preliminary SUSY analyses and coded the speech from the therapy videos. All authors revised the article critically for important intellectual content and approved the submitted version to be published.

## Conflict of Interest

The authors declare that the research was conducted in the absence of any commercial or financial relationships that could be construed as a potential conflict of interest.

## Publisher’s Note

All claims expressed in this article are solely those of the authors and do not necessarily represent those of their affiliated organizations, or those of the publisher, the editors and the reviewers. Any product that may be evaluated in this article, or claim that may be made by its manufacturer, is not guaranteed or endorsed by the publisher.

## Footnotes

1
https://www.embodiment.ch/

## References

[B1] Altmann U. Brümmel M. Meier J. Strauss B. (2021). Movement synchrony and facial synchrony as diagnostic features of depression. *J. Nerv. Mental Dis.* 209 128–136. 10.1097/NMD.0000000000001268 33214386

[B2] Altmann U. Schoenherr D. Paulick J. Deisenhofer A. K. Schwartz B. Rubel J. A. (2020). Associations between movement synchrony and outcome in patients with social anxiety disorder: evidence for treatment specific effects. *Psychother. Res.* 30 574–590. 10.1080/10503307.2019.1630779 31213149

[B3] Ardizzi M. Calbi M. Tavaglione S. Umiltà M. A. Gallese V. (2020). Audience spontaneous entrainment during the collective enjoyment of live performances: physiological and behavioral measurements. *Sci. Rep.* 10 1–12. 10.1038/s41598-020-60832-7 32123246 PMC7052145

[B4] Bänninger-Huber E. Widmer C. (1999). Affective relationship patterns and psychotherapeutic change. *Psychother. Res.* 9 74–87. 10.1093/ptr/9.1.74

[B5] Bartle-Haring S. Knerr M. Adkins K. Delaney R. O. Gangamma R. Glebova T. (2012). Trajectories of therapeutic alliance in couple versus individual therapy: three-level models. *J. Sex Marital Ther.* 38 79–107. 10.1080/0092623X.2011.569635 22268983

[B6] Bernieri F. J. (1988). Coordinated movement and rapport in teacher-student interactions. *J. Nonverb. Behav.* 12 120–138. 10.1007/bf00986930

[B7] Bordin E. S. (1979). The generalizability of the psychoanalytic concept of the working alliance. *Psychother. Theory Res. Pract.* 16 252–260. 10.1037/h0085885

[B8] Boucsein W. (2012). *Electrodermal Activity.* Berlin: Springer Science & Business Media.

[B9] Brehm J. W. (1999). The intensity of emotion. *Pers. Soc. Psychol. Rev.* 3 2–22. 15647145 10.1207/s15327957pspr0301_1

[B10] Butler E. A. (2015). Interpersonal affect dynamics: it takes two (and time) to tango. *Emot. Rev.* 7 336–341. 10.1177/1754073915590622

[B11] Cao Z. Simon T. Wei S. E. Sheikh Y. (2017). “Realtime multi-person 2d pose estimation using part affinity fields,” in *Proceedings of the IEEE Conference on Computer Vision and Pattern Recognition*, Computer Vision Foundation 7291–7299. Available online at: https://openaccess.thecvf.com/content_cvpr_2017/html/Cao_Realtime_Multi-Person_2D_CVPR_2017_paper.html

[B12] Cirelli L. K. Einarson K. M. Trainor L. J. (2014). Interpersonal synchrony increases prosocial behavior in infants. *Dev. Sci.* 17 1003–1011. 10.1111/desc.12193 25513669

[B13] Codrons E. Bernardi N. F. Vandoni M. Bernardi L. (2014). Spontaneous group synchronization of movements and respiratory rhythms. *PLoS One* 9:e107538. 10.1371/journal.pone.0107538 25216280 PMC4162643

[B14] Coutinho J. Oliveira-Silva P. Fernandes E. Gonçalves O. F. Correia D. Tschacher W. (2019). Psychophysiological synchrony during verbal interaction in romantic relationships. *Fam. Process* 58 716–733. 10.1111/famp.12371 29888517

[B15] Coutinho J. Pereira A. Oliveira-Silva P. Meier D. Lourenço V. Tschacher W. (2021). When our hearts beat together: cardiac synchrony as an entry point to understand dyadic co-regulation in couples. *Psychophysiology* 58:e13739. 10.1111/psyp.13739 33355941

[B16] Dale R. Bryant G. A. Manson J. H. Gervais M. M. (2020). Body synchrony in triadic interaction. *R. Soc. Open Sci.* 7:200095. 10.1098/rsos.200095 33047010 PMC7540751

[B17] Delaherche E. Chetouani M. Mahdhaoui A. Saint-Georges C. Viaux S. Cohen D. (2012). Interpersonal synchrony: a survey of evaluation methods across disciplines. *IEEE Trans. Affect. Comput.* 3 349–365. 10.1109/t-affc.2012.12

[B18] Deres-Cohen K. Lipsitz-Odess I. Fisher H. Ramseyer F. T. Lutz W. Zilcha-Mano S. (2021). Shedding light on the effects of supportive techniques on nonverbal synchrony and their moderators in psychotherapy for depression. *Psychother. Res.* 1–14. 10.1080/10503307.2021.1966542 34445938

[B19] Feldman R. (2017). The neurobiology of human attachments. *Trends Cogn. Sci.* 21 80–99. 10.1016/j.tics.2016.11.007 28041836

[B20] Ferguson N. (1977). Simultaneous speech, interruptions and dominance. *Br. J. Soc. Clin. Psychol.* 16 295–302. 10.1111/j.2044-8260.1977.tb00235.x

[B21] Finset A. (2014). Talk-in-interaction and neuropsychological processes. *Scand. J. Psychol.* 55 212–218. 10.1111/sjop.12127 24758614

[B22] Fowles D. C. (1980). The three arousal model: implications of Gray’s two-factor learning theory for heart rate, electrodermal activity, and psychopathy. *Psychophysiology* 17 87–104. 10.1111/j.1469-8986.1980.tb00117.x 6103567

[B23] Fredrikson M. Furmark T. Olsson M. T. Fischer H. Andersson J. Långström B. (1998). Functional neuroanatomical correlates of electrodermal activity: a positron emission tomographic study. *Psychophysiology* 35 179–185. 9529944

[B24] Friedlander M. L. Escudero V. Heatherington L. (2006b). *Therapeutic Alliances in Couple and Family Therapy: An Empirically Informed Guide to Practice.* Washington, DC: American Psychological Association.

[B25] Friedlander M. L. Escudero V. Horvath A. O. Heatherington L. Cabero A. Martens M. P. (2006a). System for observing family therapy alliances: a tool for research and practice. *J. Counsel. Psychol.* 53 214–225.

[B26] Fujiwara K. Daibo I. (2021). Empathic accuracy and interpersonal coordination: behavior matching can enhance accuracy but interactional synchrony may not. *J. Soc. Psychol.* 1–18. 10.1080/00224545.2021.1983509 34651552

[B27] Fujiwara K. Yokomitsu K. (2021). Video-based tracking approach for nonverbal synchrony: a comparison of Motion Energy Analysis and OpenPose. *Behav. Res. Methods* 53 2700–2711. 10.3758/s13428-021-01612-7 34027597

[B28] Galbusera L. Finn M. T. Tschacher W. Kyselo M. (2019). Interpersonal synchrony feels good but impedes self-regulation of affect. *Sci. Rep.* 9 1–12. 10.1038/s41598-019-50960-0 31604966 PMC6789117

[B29] Gaume J. Hallgren K. A. Clair C. Schmid Mast M. Carrard V. Atkins D. C. (2019). Modeling empathy as synchrony in clinician and patient vocally encoded emotional arousal: a failure to replicate. *J. Counsel. Psychol.* 66 341–350. 10.1037/cou0000322 30702323 PMC7286050

[B30] Gottman J. M. Coan J. Carrere S. Swanson C. (1998). Predicting marital happiness and stability from newlywed interactions. *J. Mar. Fam.* 60 5–22.

[B31] Hagoort P. Levelt W. J. (2009). The speaking brain. *Science* 326 372–373. 19833945 10.1126/science.1181675

[B32] Hall J. A. Harrigan J. A. Rosenthal R. (1995). Nonverbal behavior in clinician-patient interaction. *Appl. Prev. Psychol.* 4 21–37. 10.1016/s0962-1849(05)80049-6

[B33] Harrigan J. A. Oxman T. E. Rosenthal R. (1985). Rapport expressed through nonverbal behavior. *J. Nonverb. Behav.* 9 95–110. 10.1007/bf00987141

[B34] Hatfield E. Rapson R. L. Le Y. C. (2009). *Emotional Contagion and Empathy.* Cambridge, MA: MIT.

[B35] Helm J. L. Sbarra D. Ferrer E. (2012). Assessing cross-partner associations in physiological responses via coupled oscillator models. *Emotion* 12 748–762. 10.1037/a0025036 21910541

[B36] Hove M. J. Risen J. L. (2009). It’s all in the timing: interpersonal synchrony increases affiliation. *Soc. Cogn.* 27 949–960.

[B37] Imel Z. E. Barco J. S. Brown H. J. Baucom B. R. Baer J. S. Kircher J. C. (2014). The association of therapist empathy and synchrony in vocally encoded arousal. *J. Counsel. Psychol.* 61 146–153. 10.1037/a0034943 24274679 PMC4133554

[B38] Isosävi J. (2020). Cultural outsiders’ reported adherence to Finnish and French politeness norms. *J. Pragm.* 155 177–192.

[B39] Johnson L. N. Wright D. W. (2002). Revisiting Bordin’s theory on the therapeutic alliance: implications for family therapy. *Contemp. Fam. Ther.* 24 257–269.

[B40] Karvonen A. (2017). Sympathetic nervous system synchrony between participants of couple therapy. *Jyväskylä Stud. Educ. Psychol. Soc. Res.* 599 38–41.

[B41] Karvonen A. Kykyri V. L. Kaartinen J. Penttonen M. Seikkula J. (2016). Sympathetic nervous system synchrony in couple therapy. *J. Marital Fam. Ther.* 42 383–395. 26748869 10.1111/jmft.12152

[B42] Kita S. Alibali M. W. Chu M. (2017). How do gestures influence thinking and speaking? The gesture-for-conceptualization hypothesis. *Psychol. Rev.* 124 245–266. 10.1037/rev0000059 28240923

[B43] Konvalinka I. Xygalatas D. Bulbulia J. Schjødt U. Jegindø E. M. Wallot S. (2011). Synchronized arousal between performers and related spectators in a fire-walking ritual. *Proc. Natl. Acad. Sci. U.S.A.* 108 8514–8519. 10.1073/pnas.1016955108 21536887 PMC3100954

[B44] Koole S. L. Tschacher W. (2016). Synchrony in psychotherapy: a review and an integrative framework for the therapeutic alliance. *Front. Psychol.* 7:862. 10.3389/fpsyg.2016.00862 27378968 PMC4907088

[B45] Kreibig S. D. (2010). Autonomic nervous system activity in emotion: a review. *Biol. Psychol.* 84 394–421. 10.1016/j.biopsycho.2010.03.010 20371374

[B46] Kykyri V. L. Tourunen A. Nyman-Salonen P. Kurri K. Wahlström J. Kaartinen J. (2019). Alliance formations in couple therapy–a multi-modal and multi-method study. *J. Couple Relationsh. Ther.* 18 189–222. 10.1080/15332691.2018.1551166

[B47] Lakens D. Stel M. (2011). If they move in sync, they must feel in sync: movement synchrony leads to attributions of rapport and entitativity. *Soc. Cogn.* 29 1–14.

[B48] Lausberg H. Kita S. (2003). The content of the message influences the hand choice in co-speech gestures and in gesturing without speaking. *Brain Lang.* 86 57–69. 10.1016/s0093-934x(02)00534-5 12821415

[B49] Levenson R. W. Gottman J. M. (1983). Marital interaction: physiological linkage and affective exchange. *J. Pers. Soc. Psychol.* 45 587–597. 10.1037//0022-3514.45.3.587 6620126

[B50] Lutz W. Prinz J. N. Schwartz B. Paulick J. Schoenherr D. Deisenhofer A. K. (2020). Patterns of early change in interpersonal problems and their relationship to nonverbal synchrony and multidimensional outcome. *J. Counsel. Psychol.* 67 449–461. 10.1037/cou0000376 32614226

[B51] Macrae C. N. Duffy O. K. Miles L. K. Lawrence J. (2008). A case of hand waving: action synchrony and person perception. *Cognition* 109 152–156. 10.1016/j.cognition.2008.07.007 18755450

[B52] Marci C. D. Ham J. Moran E. Orr S. P. (2007). Physiologic correlates of perceived therapist empathy and social-emotional process during psychotherapy. *J. Nerv. Ment. Dis.* 195 103–111. 10.1097/01.nmd.0000253731.71025.fc 17299296

[B53] Marci C. D. Orr S. P. (2006). The effect of emotional distance on psychophysiologic concordance and perceived empathy between patient and interviewer. *Appl. Psychophysiol. Biofeedback* 31 115–128. 10.1007/s10484-006-9008-4 16724278

[B54] Mayo O. Gordon I. (2020). In and out of synchrony—behavioral and physiological dynamics of dyadic interpersonal coordination. *Psychophysiology* 57:e13574. 10.1111/psyp.13574 32221984

[B55] Mende M. A. Schmidt H. (2021). Psychotherapy in the framework of embodied cognition—does interpersonal synchrony influence therapy success? *Front. Psychiatry* 12:325. 10.3389/fpsyt.2021.562490 PMC8019827 33828491

[B56] Messina I. Palmieri A. Sambin M. Kleinbub J. R. Voci A. Calvo V. (2013). Somatic underpinnings of perceived empathy: the importance of psychotherapy training. *Psychother. Res.* 23 169–177. 10.1080/10503307.2012.748940 23234457

[B57] Miles L. K. Nind L. K. Henderson Z. Macrae C. N. (2010). Moving memories: behavioral synchrony and memory for self and others. *J. Exp. Soc. Psychol.* 46 457–460.

[B58] Miles L. K. Nind L. K. Macrae C. N. (2009). The rhythm of rapport: interpersonal synchrony and social perception. *J. Exp. Soc. Psychol.* 45 585–589. 10.1016/j.jesp.2009.02.002

[B59] Mogan R. Fischer R. Bulbulia J. A. (2017). To be in synchrony or not? A meta-analysis of synchrony’s effects on behavior, perception, cognition and affect. *J. Exp. Soc. Psychol.* 72 13–20.

[B60] Montague P. R. Berns G. S. Cohen J. D. McClure S. M. Pagnoni G. Dhamala M. (2002). Hyperscanning: simultaneous fMRI during linked social interactions. *Neuroimage* 16 1159–1164. 10.1006/nimg.2002.1150 12202103

[B61] Noy L. Levit-Binun N. Golland Y. (2015). Being in the zone: physiological markers of togetherness in joint improvisation. *Front. Hum. Neurosci.* 9:187. 10.3389/fnhum.2015.00187 25999832 PMC4419713

[B62] Nummenmaa L. Glerean E. Viinikainen M. Jääskeläinen I. P. Hari R. Sams M. (2012). Emotions promote social interaction by synchronizing brain activity across individuals. *Proc. Natl. Acad. Sci. U.S.A.* 109 9599–9604. 10.1073/pnas.1206095109 22623534 PMC3386135

[B63] Nyman-Salonen P. Kykyri V.-L. Tschacher W. Muotka J. Tourunen A. Penttonen M. (2021b). Nonverbal synchrony in couple therapy linked to the clients’ wellbeing and the therapeutic alliance. *Front. Psychol.* 12:718353. 10.3389/fpsyg.2021.718353 34858258 PMC8631962

[B64] Nyman-Salonen P. Tourunen A. Kykyri V. L. Penttonen M. Kaartinen J. Seikkula J. (2021a). Studying nonverbal synchrony in couple therapy—observing implicit posture and movement synchrony. *Contemp. Fam. Ther.* 43 69–87. 10.1007/s10591-020-09555-5

[B65] Orlinsky D. E. Rønnestad M. H. Willutzki U. (2004). “Fifty years of psychotherapy process-outcome research: continuity and change,” in *Bergin and Garfield’s handbook of Psychotherapy and Behavior Change*, ed. Lambert M. J. (New York, NY: Wiley), 307–389.

[B66] Palmieri A. Kleinbub J. R. Calvo V. Benelli E. Messina I. Sambin M. (2018). Attachment-security prime effect on skin-conductance synchronization in psychotherapists: an empirical study. *J. Counsel. Psychol.* 65 490–499. 10.1037/cou0000273 29494169

[B67] Palumbo R. V. Marraccini M. E. Weyandt L. L. Wilder-Smith O. McGee H. A. Liu S. (2017). Interpersonal autonomic physiology: a systematic review of the literature. *Pers. Soc. Psychol. Rev.* 21 99–141. 10.1177/1088868316628405 26921410

[B68] Paulick J. Deisenhofer A. K. Ramseyer F. Tschacher W. Boyle K. Rubel J. (2018a). Nonverbal synchrony: a new approach to better understand psychotherapeutic processes and drop-out. *J. Psychother. Integr.* 28 367–384.

[B69] Paulick J. Rubel J. A. Deisenhofer A. K. Schwartz B. Thielemann D. Altmann U. (2018b). Diagnostic features of nonverbal synchrony in psychotherapy: comparing depression and anxiety. *Cogn. Ther. Res.* 42 539–551.

[B70] Pfänder S. Couper-Kuhlen E. (2019). Turn-sharing revisited: an exploration of simultaneous speech in interactions between couples. *J. Pragm.* 147 22–48. 10.1016/j.pragma.2019.05.010

[B71] Prinz J. Boyle K. Ramseyer F. Kabus W. Bar-Kalifa E. Lutz W. (2021). Within and between associations of nonverbal synchrony in relation to Grawe’s general mechanisms of change. *Clin. Psychol. Psychother.* 28 159–168. 10.1002/cpp.2498 32794374

[B72] Ramseyer F. Tschacher W. (2008). “Synchrony in dyadic psychotherapy sessions,” in *Simultaneity: Temporal Structures and Observer Perspectives*, eds Susie V. Otto E. R. Marks-Tarlow T. (Singapore: World Scientific), 329–347.

[B73] Ramseyer F. Tschacher W. (2011). Nonverbal synchrony in psychotherapy: coordinated body movement reflects relationship quality and outcome. *J. Consult. Clin. Psychol.* 79 284–295. 10.1037/a0023419 21639608

[B74] Ramseyer F. Tschacher W. (2014). Nonverbal synchrony of head-and body-movement in psychotherapy: different signals have different associations with outcome. *Front. Psychol.* 5:979. 10.3389/fpsyg.2014.00979 25249994 PMC4155778

[B75] Ramseyer F. T. (2020b). Exploring the evolution of nonverbal synchrony in psychotherapy: the idiographic perspective provides a different picture. *Psychother. Res.* 30 622–634. 10.1080/10503307.2019.1676932 31603387

[B76] Ramseyer F. T. (2020a). Motion Energy Analysis (MEA). a primer on the assessment of motion from video. *J. Counsel. Psychol.* 67 536–549. 10.1037/cou0000407 32614233

[B77] Reich C. M. Berman J. S. Dale R. Levitt H. M. (2014). Vocal synchrony in psychotherapy. *J. Soc. Clin. Psychol.* 33 481–494. 10.1521/jscp.2014.33.5.481

[B78] Rennung M. Göritz A. S. (2016). Prosocial consequences of interpersonal synchrony. A meta-analysis. *Zeitschrift für Psychologie* 224 168–189. 10.1027/2151-2604/a000252 28105388 PMC5137339

[B79] Safran J. D. Muran J. C. (2006). Has the concept of the therapeutic alliance outlived its usefulness? *Psychother. Theory Res. Pract. Train.* 43 286–291. 10.1037/0033-3204.43.3.286 22122099

[B80] Scheidt C. E. Pfänder S. Ballati A. Schmidt S. Lahmann C. (2021). Language and movement synchronization in dyadic psychotherapeutic interaction–a qualitative review and a proposal for a classification. *Front. Psychol.* 12:696448. 10.3389/fpsyg.2021.696448 34744862 PMC8569105

[B81] Schipke J. D. Arnold G. Pelzer M. (1999). Effect of respiration rate on short-term heart rate variability. *J. Clin. Basic Cardiol.* 2 92–95.

[B82] Schoenherr D. Paulick J. Strauss B. M. Deisenhofer A. K. Schwartz B. Rubel J. A. (2019). Nonverbal synchrony predicts premature termination of psychotherapy for social anxiety disorder. *Psychotherapy* 56 503–513. 10.1037/pst0000216 30869972

[B83] Schoenherr D. Strauss B. Paulick J. Deisenhofer A. K. Schwartz B. Rubel J. A. (2021). Movement synchrony and attachment related anxiety and avoidance in social anxiety disorder. *J. Psychother. Integr.* 31 163–179. 10.1037/int0000187

[B84] Seikkula J. Karvonen A. Kykyri V. L. Kaartinen J. Penttonen M. (2015). The embodied attunement of therapists and a couple within dialogical psychotherapy: an introduction to the relational mind research project. *Fam. Process* 54 703–715. 10.1111/famp.12152 25810020

[B85] Seikkula J. Olson M. (2016). “Therapists’ responses for enhancing change through dialogue: dialogical investigations of change,” in *Research Perspectives in Couple Therapy* eds Borcsa M. Rober P. (Cham: Springer), 47–69. 10.1007/978-3-319-23306-2_5

[B86] Slovák P. Tennent P. Reeves S. Fitzpatrick G. (2014). “Exploring skin conductance synchronisation in everyday interactions,” in *Proceedings of the 8th Nordic Conference on Human-Computer Interaction: Fun, Fast, Foundational* (New York, NY: ACM), 511–520.

[B87] Stein P. K. Bosner M. S. Kleiger R. E. Conger B. M. (1994). Heart rate variability: a measure of cardiac autonomic tone. *Am. Heart J.* 127 1376–1381. 8172068 10.1016/0002-8703(94)90059-0

[B88] Sullivan G. M. Feinn R. (2012). Using effect size—or why the P value is not enough. *J. Grad. Med. Educ.* 4 279–282. 10.4300/JGME-D-12-00156.1 23997866 PMC3444174

[B89] Timmons A. C. Margolin G. Saxbe D. E. (2015). Physiological linkage in couples and its implications for individual and interpersonal functioning: a literature review. *J. Fam. Psychol.* 29 720–731. 10.1037/fam0000115 26147932 PMC4593729

[B90] Tourunen A. Kykyri V. L. Seikkula J. Kaartinen J. Tolvanen A. Penttonen M. (2020). Sympathetic nervous system synchrony: an exploratory study of its relationship with the therapeutic alliance and outcome in couple therapy. *Psychotherapy* 57 160–173. 10.1037/pst0000198 30667244

[B91] Tschacher W. Greenwood S. Egermann H. Wald-Fuhrmann M. Czepiel A. Tröndle M. (2021). Physiological synchrony in audiences of live concerts. *Psychol. Aesthet. Creat. Arts*, Advance online publication. 10.1038/s41598-021-00492-3 34789746 PMC8599424

[B92] Tschacher W. Haken H. (2019). *The Process of Psychotherapy: Causation and Chance.* Berlin: Springer.

[B93] Tschacher W. Haken H. (2020). Causation and chance: detection of deterministic and stochastic ingredients in psychotherapy processes. *Psychother. Res.* 30 1075–1087. 10.1080/10503307.2019.1685139 31690229

[B94] Tschacher W. Meier D. (2020). Physiological synchrony in psychotherapy sessions. *Psychother. Res.* 30 558–573. 10.1080/10503307.2019.1612114 31060474

[B95] van Baaren R. B. Holland R. W. Kawakami K. Van Knippenberg A. (2004). Mimicry and prosocial behavior. *Psychol. Sci.* 15 71–74. 10.1111/j.0963-7214.2004.01501012.x 14717835

[B96] Wilson M. (2002). Six views of embodied cognition. *Psychon. Bull. Rev.* 9 625–636. 10.3758/bf03196322 12613670

[B97] Wiltshire T. J. Philipsen J. S. Trasmundi S. B. Jensen T. W. Steffensen S. V. (2020). Interpersonal coordination dynamics in psychotherapy: a systematic review. *Cogn. Ther. Res.* 44 752–773. 10.3389/fpsyg.2017.02053 29225589 PMC5705546

[B98] Zhang M. Dumas G. Kelso J. S. Tognoli E. (2016). Enhanced emotional responses during social coordination with a virtual partner. *Int. J. Psychophysiol.* 104 33–43. 10.1016/j.ijpsycho.2016.04.001 27094374 PMC4899205

